# A new era in the treatment of metabolic dysfunction-associated steatotic liver disease (MASLD): clinical breakthroughs and challenges

**DOI:** 10.3389/fphar.2026.1815576

**Published:** 2026-08-31

**Authors:** Zhifu You, Linglong Yang, Yuanhao Long, Dongye You, Qian Zhao

**Affiliations:** 1 Department I of Gastroenterology, First Affiliated Hospital of Heilongjiang University of Chinese Medicine, Harbin, China; 2 Department of Rehabilitation Medicine, South Hospital of Qiqihar Traditional Chinese Medicine Hospital, Qiqihar, China

**Keywords:** GLP-1 receptor agonist, MASH, MASLD, metabolic dysfunction-associated steatotic liver disease, pharmacologic therapy

## Abstract

Metabolic dysfunction-associated steatotic liver disease (MASLD), affecting nearly one-third of adults worldwide, encompasses a spectrum from steatosis to steatohepatitis (MASH), advanced fibrosis, cirrhosis, and hepatocellular carcinoma, with fibrosis stage as the primary determinant of liver-related outcomes and cardiovascular disease as the leading cause of mortality. Despite robust evidence that lifestyle interventions achieving ≥10% weight loss induce fibrosis regression in many patients, sustained success in routine practice remains elusive—realised in fewer than 15% of individuals—exposing a critical efficacy-effectiveness gap that demands pharmacologic therapy for those with clinically significant fibrosis. The period 2024–2026 has marked a transformative shift, with resmetirom (thyroid hormone receptor-β agonist) gaining accelerated approval in 2024 as the first agent to meet histologic endpoints in MASH, followed by semaglutide (GLP-1 receptor agonist) in 2025, both demonstrating substantial MASH resolution and fibrosis improvement in phase 3 trials; a diverse pipeline, including tirzepatide, efruxifermin, lanifibranor, denifanstat, and dual-incretin agonists, has yielded promising histologic and metabolic outcomes, with several advancing to phase 3 and updated guidelines endorsing pharmacotherapy for F2–F3 MASH, prioritising comorbidity-guided selection and synergy with lifestyle modification. Yet, these histologic gains must now be translated into reduced morbidity and mortality through an ambitious agenda of long-term outcome trials, precision biomarkers, and equitable, multidisciplinary care models.

## Introduction

Metabolic dysfunction-associated steatotic liver disease (MASLD), encompassing a spectrum from simple steatosis (MASL) to metabolic dysfunction-associated steatohepatitis (MASH), fibrosis, cirrhosis, and hepatocellular carcinoma, affects approximately 30% of adults globally and represents a leading cause of liver-related morbidity (Younossi et al., 2023; Riazi et al., 2022). Central to this spectrum is the stage of liver fibrosis (F), a histologically defined assessment of scar tissue accumulation that serves as the primary driver of clinical outcomes. The F0 to F4 fibrosis staging system, derived from the NASH Clinical Research Network (NASH CRN) classification, provides the foundational framework for risk stratification, guiding all major therapeutic decisions and forming the basis for patient selection in pivotal registration trials. Progression to advanced fibrosis occurs in up to 30% of cases, with MASH and fibrosis stage being the primary determinants of liver-related outcomes. Critically, cardiovascular events account for approximately 40% of deaths in this population, highlighting the systemic metabolic dysfunction at the core of the condition (Targher et al., 2024; Mantovani et al., 2020). For decades, management relied exclusively on lifestyle modification, with off-label agents such as vitamin E or pioglitazone providing modest and histologically inconsistent benefits accompanied by safety concerns.

Lifestyle modification remains the cornerstone of MASLD management, supported by a robust dose-response relationship between weight loss and histologic improvement. Reductions of ≥5% body weight typically resolve steatosis, ≥7% improve inflammatory activity, and ≥10% achieve fibrosis regression in a substantial proportion of patients (EAftSotLE and EASDEurope an Association for the Study of Diabetes EASDEuropean Association for the Study of Obesity EASO, 2024). Dietary composition further enhances outcomes independent of weight loss; Mediterranean patterns rich in unsaturated fats and polyphenols improve insulin sensitivity and reduce hepatic *de novo* lipogenesis, while regular aerobic or resistance exercise decreases intrahepatic triglycerides and cardiometabolic risk through partially weight-independent mechanisms.

Despite this strong evidence base, real-world effectiveness is limited. Sustained ≥10% weight loss is achieved in fewer than 15% of patients in routine practice, with high rates of weight regain over time, as evidenced by large registries such as TARGET-NASH (Barritt et al., 2017). This efficacy-effectiveness gap underscores the critical need for pharmacologic intervention in patients with clinically significant fibrosis.

The years 2024–2026 have ushered in a transformative era in MASLD therapeutics. Resmetirom, a liver-selective thyroid hormone receptor-β agonist, received accelerated FDA approval in March 2024 based on the pivotal MAESTRO-NASH trial, becoming the first agent to meet stringent histologic endpoints in MASH (Harrison et al., 2024). Semaglutide followed with approval in 2025 after the phase 3 ESSENCE trial demonstrated superior resolution of MASH and fibrosis improvement (Sanyal et al., 2025). A robust pipeline has advanced in parallel: tirzepatide (GLP-1/GIP dual agonist) has shown compelling phase 2b data with phase 3 ongoing (Caussy et al., 2025; Loomba et al., 2024a); efruxifermin (FGF21 analogue) has reported sustained fibrosis benefits at 96 weeks and signals in compensated cirrhosis (Noureddin et al., 2025a; Noureddin et al., 2025b); lanifibranor (pan-PPAR agonist) is anticipated to report phase 3 topline results in 2026 (Barb et al., 2025; Boursier et al., 2025); denifanstat (FASN inhibitor) has entered phase 3 (Loomba et al., 2024b); and novel incretin-based agents such as pemvidutide and survodutide have yielded encouraging phase 2b histologic and metabolic outcomes (Noureddin et al., 2025c; Harrison et al., 2025a; Sanyal et al., 2024). Updated AASLD and EASL guidelines now incorporate pharmacotherapy for patients with biopsy-confirmed or high-probability F2–F3 MASH, particularly those with cardiometabolic comorbidities (Bansal et al., 2026).

Importantly, emerging data reinforce that pharmacotherapy enhances rather than replaces lifestyle efforts. Many novel agents—particularly GLP-1-based therapies—facilitate clinically meaningful weight loss (10%–15%), thereby improving adherence and overcoming physiological barriers to sustained behavioral change. Secondary analyses from MAESTRO-NASH demonstrate that patients achieving ≥5% weight loss on resmetirom had markedly higher rates of MASH resolution (56.6% vs. 33.8%) and fibrosis improvement (40.6% vs. 31.5%), with greater reductions in MRI-PDFF and liver stiffness (Noureddin et al., 2025d). This synergy positions lifestyle intervention as the essential foundation and pharmacotherapy as a powerful enhancer, enabling more effective disease modification in high-risk individuals.

This perspective examines these breakthroughs from a clinical hepatology viewpoint, focusing on practical implementation of pharmacotherapy, evolving treatment algorithms, patient selection, treatment optimization, monitoring, and persistent challenges, including the future of combination therapy, precision medicine, and management of extrahepatic outcomes. By integrating lifestyle as the non-negotiable base with new pharmacologic tools, the field has shifted from “whether to treat” to “how best to optimize treatment” for patients at highest risk.

## Pharmacologic therapies: from clinical trials to clinical practice

### Approved agents

#### Resmetirom (THR-β agonist)

Resmetirom, a liver-selective thyroid hormone receptor-β agonist, targets intrahepatic lipotoxicity, inflammation, and fibrogenesis through modulation of mitochondrial function and lipid metabolism. In the pivotal 52-week MAESTRO-NASH trial enrolling patients with biopsy-confirmed MASH and fibrosis stages F1B–F3, resmetirom 100 mg daily achieved the dual primary endpoints: MASH resolution without fibrosis worsening in 29.9% versus 9.7% with placebo (difference 20.2%, 95% CI 13.2%–27.2%; p < 0.001), and ≥1-stage fibrosis improvement without MASH worsening in 25.9% versus 14.2% with placebo (difference 11.7%, 95% CI 4.8%–18.6%; p < 0.001) (Harrison et al., 2024). Secondary analyses confirmed efficacy irrespective of background therapy with GLP-1 receptor agonists or SGLT2 inhibitors, with enhanced responses in patients achieving concurrent weight loss (Noureddin et al., 2025d). Importantly, resmetirom provides significant LDL-cholesterol reduction (approximately 15–20 mg/dL), offering additive cardiovascular risk modification—a valuable attribute given that cardiovascular events represent the leading cause of mortality in this population. Approved in 2024 for F2–F3 MASH, resmetirom clinical use requires attention to gastrointestinal tolerability (diarrhea, nausea occurring in 20%–30%, generally mild to moderate), thyroid function monitoring (given theoretical THR-β effects), and drug-drug interactions (primarily CYP2C8 substrates). Decompensated cirrhosis (Child-Pugh B/C) is contraindicated based on safety data.

#### Semaglutide (GLP-1 receptor agonist)

Semaglutide 2.4 mg weekly, evaluated in the phase 3 ESSENCE trial involving 1,197 patients with F2–F3 MASH, demonstrated histologic efficacy at the 72-week interim analysis. MASH resolution without fibrosis worsening occurred in 62.9% of semaglutide-treated patients versus 34.3% with placebo (estimated difference 28.7%, 95% CI 21.1%–36.2%; p < 0.001) (Sanyal et al., 2025). Fibrosis improvement by ≥ 1 stage without MASH worsening was achieved in 36.8% versus 22.4%, respectively (difference 14.4%, 95% CI 7.5%–21.3%; p < 0.001). Mean weight loss reached 10.5% with semaglutide versus 2.0% with placebo, and combined histologic improvement (both MASH resolution and fibrosis reduction) occurred in 32.7% versus 16.1% (Sanyal et al., 2025). Approved in 2025, semaglutide is recommended for patients with moderate-to-advanced fibrosis, particularly those with comorbid T2DM or obesity (Bansal et al., 2026). Gastrointestinal adverse events (nausea, diarrhea, vomiting) affect approximately 60%–70% of patients but are manageable with gradual dose titration. Contraindications include personal or family history of medullary thyroid carcinoma and multiple endocrine neoplasia syndrome type 2.

### Pipeline agents with phase 3 potential

Across the pipeline, gastrointestinal adverse events remain the most common class effect of incretin-based therapies, generally mild-to-moderate and mitigated by dose titration, whereas non-incretin agents (e.g., lanifibranor, denifanstat) exhibit more mechanism-specific tolerability profiles.

#### Tirzepatide (GLP-1/GIP dual agonist)

In the phase 2 SYNERGY-NASH trial enrolling 190 patients with F2–F3 MASH, tirzepatide (5 mg, 10 mg, or 15 mg weekly) demonstrated dose-dependent histologic efficacy at 52 weeks. MASH resolution without fibrosis worsening was achieved in 44%, 56%, and 62% of patients receiving 5 mg, 10 mg, and 15 mg, respectively, compared to 10% with placebo (p < 0.001 for all comparisons) (Loomba et al., 2024a). Fibrosis improvement by ≥ 1 stage without MASH worsening occurred in approximately 51%–55% across tirzepatide doses versus 30% with placebo. Weight loss reached 15%–17% at the highest dose, and *post hoc* mechanistic analyses linked histologic response to normalization of liver fat (MRI-PDFF <5%) and improvement in adipose tissue insulin sensitivity (Caussy et al., 2025). Phase 3 trials are actively enrolling. Its safety profile mirrors that observed in large T2DM/obesity trials, dominated by dose-dependent gastrointestinal adverse events (nausea, diarrhea, vomiting, primarily during dose escalation; discontinuation rates ∼5–8%). No new safety signals specific to MASH were identified. If approved, tirzepatide would offer the most potent weight loss and among the highest histologic response rates observed to date. Its safety profile in MASH aligns with its established use in diabetes and obesity, characterized by dose-dependent gastrointestinal adverse events (e.g., nausea, diarrhea, vomiting), which can be effectively mitigated through a gradual dose-escalation protocol.

#### Efruxifermin (FGF21 analogue)

Fibroblast growth factor 21 (FGF21) analogues target multiple metabolic pathways including insulin sensitivity, energy expenditure, and hepatic lipid metabolism. In the phase 2b HARMONY trial (F2–F3 MASH, n = 128), efruxifermin 50 mg weekly achieved ≥1-stage fibrosis improvement without MASH worsening in 49% of patients versus 19% with placebo at 96 weeks (difference 31%, 95% CI 12%–49%; p = 0.003) (Noureddin et al., 2025a). MASH resolution without fibrosis worsening at 96 weeks was observed in 57% with 50 mg efruxifermin versus 15% with placebo. In the phase 2b SYMMETRY trial specifically enrolling patients with compensated MASH cirrhosis (F4), fibrosis improvement without MASH worsening at 96 weeks occurred in 29% receiving efruxifermin 50 mg versus 11% with placebo, suggesting potential benefit in this difficult-to-treat population (Noureddin et al., 2025b). Combination with GLP-1 receptor agonists demonstrated additive hepatic fat reduction without unexpected safety signals (Harrison et al., 2025b). Gastrointestinal adverse events (nausea, diarrhea) are common but generally mild to moderate. Phase 3 development is underway.

#### Lanifibranor (Pan-PPAR agonist)

Lanifibranor activates all three-peroxisome proliferator-activated receptor isoforms (PPAR-α, -γ, -δ), comprehensively targeting insulin resistance, inflammation, and fibrogenesis. In a mechanistic phase 2 study of patients with T2DM and MASLD, lanifibranor 800 mg daily for 24 weeks reduced intrahepatic triglycerides by approximately 50% (vs. 12% with placebo, p < 0.01) and improved hepatic, muscle, and adipose tissue insulin sensitivity measured by gold-standard hyperinsulinemic-euglycemic clamp (Barb et al., 2025). Adiponectin increased 2.4-fold, and cardiometabolic parameters including HbA1c and HDL-cholesterol improved significantly. Modest weight gain (+2.7%) was observed. Subsequent biomarker analyses identified baseline adiponectin, ferritin, and dynamic changes in matrix metalloproteinase 9, hyaluronic acid, and aminotransferases that predicted histologic response, offering potential for precision prescribing (Boursier et al., 2025). The phase 3 NATiV3 trial in non-cirrhotic MASH is expected to report topline results in 2026. In clinical studies, lanifibranor was generally well-tolerated, with the most notable adverse event being modest weight gain (+2.7%), a class effect of PPAR-γ activation. This feature, while undesirable in an obesity-centric disease, is manageable and will require careful consideration during patient selection and monitoring, particularly favoring those without severe concurrent obesity.

#### Denifanstat (FASN inhibitor)

Denifanstat, an oral fatty acid synthase inhibitor, blocks *de novo* lipogenesis—a key pathway driving lipotoxicity and fibrogenesis. In the phase 2b FASCINATE-2 trial (F2–F3 MASH, n = 168), denifanstat 50 mg daily for 52 weeks achieved ≥2-point improvement in NAS without fibrosis worsening in 38% versus 16% with placebo (p = 0.0035), and MASH resolution with ≥2-point NAS improvement in 26% versus 11% (p = 0.017) (Loomba et al., 2024b). Fibrosis improvement by ≥ 1 stage without MASH worsening occurred in 41% versus 18%. The agent was well-tolerated; common adverse events included dry eye symptoms (9%) and alopecia (19%), all grade 1–2. Phase 3 development has been initiated.

#### Pemvidutide (GLP-1/glucagon dual agonist)

Pemvidutide combines GLP-1 receptor agonism with glucagon receptor activation, the latter directly stimulating hepatic fatty acid oxidation and inhibiting lipogenesis. In the phase 2b IMPACT trial (F2–F3 MASH, n = 212), MASH resolution without fibrosis worsening at 24 weeks was achieved in 58% (1.2 mg) and 52% (1.8 mg) of pemvidutide-treated patients versus 20% with placebo (p < 0.0001) (Noureddin et al., 2025c). Fibrosis improvement without MASH worsening at this early timepoint showed numerical trends favoring pemvidutide but did not reach statistical significance (33%–36% vs. 28%). Earlier phase 2 data demonstrated liver fat reduction up to 68.5% at 12 weeks, with 94% of patients achieving ≥30% liver fat reduction and 56% achieving normalization (≤5% liver fat) at the 1.8 mg dose (Harrison et al., 2025a). Phase 3 planning is underway.

#### Survodutide (GLP-1/glucagon dual agonist)

In the phase 2 trial of survodutide enrolling 293 patients with F1–F3 MASH, MASH improvement without fibrosis worsening at 48 weeks occurred in 47%–62% across doses versus 14% with placebo (p < 0.001) (Sanyal et al., 2024). Liver fat reduction ≥30% was achieved in 57%–67% versus 14%, and fibrosis improvement by ≥ 1 stage was observed in 34%–36% versus 22%. Gastrointestinal adverse events (nausea 66%, diarrhea 49%, vomiting 41%) were more frequent with survodutide but serious adverse events were comparable to placebo (8% vs. 7%). Phase 3 trials are ongoing.

#### ION224 (DGAT2 antisense inhibitor)

ION224, a liver-directed antisense oligonucleotide inhibiting diacylglycerol O-acyltransferase 2, suppresses *de novo* lipogenesis through a weight-independent mechanism. In the phase 2 ION224-CS2 trial (F1–F3 MASH, n = 160), ≥2-point NAS reduction with ≥1-point improvement in ballooning or lobular inflammation without fibrosis worsening at 51 weeks was achieved in 46% (90 mg) and 59% (120 mg) of patients versus 19% with placebo (p = 0.0094 and p = 0.0002, respectively) (Loomba et al., 2025). Importantly, these improvements were independent of body weight changes, supporting potential for combination with weight-loss agents. The agent was well-tolerated with no treatment-related serious adverse events.

### Clinical positioning and therapeutic selection

The expanding therapeutic landscape necessitates a stratified approach to patient management. Current evidence supports the following framework:

F2–F3 MASH: This population represents the primary target for pharmacotherapy based on highest risk of liver-related outcomes and inclusion in registration trials. Approved options include resmetirom (THR-β agonist) and semaglutide (GLP-1 RA), with tirzepatide, efruxifermin, and others anticipated pending regulatory decisions.

F0–F1 MASL: Lifestyle intervention remains primary, with pharmacotherapy reserved for high-risk individuals (e.g., those with T2DM, multiple metabolic risk factors, or rapid progression on non-invasive monitoring).

F4 Compensated Cirrhosis: Data remain limited, with most registration trials excluding this population. Emerging evidence with efruxifermin suggests potential for fibrosis improvement in compensated cirrhosis (9), but confirmatory phase 3 data are needed. Clinical decisions require careful consideration of portal hypertension status and hepatic reserve.

Comorbidity-guided selection offers a practical initial approach. Patients with predominant T2DM/obesity may preferentially receive GLP-1-based therapies (semaglutide, soon tirzepatide) addressing both hepatic and extrahepatic manifestations. Those with significant dyslipidemia may benefit from resmetirom’s LDL-lowering effects. As multiple agents become available, the critical question shifts from “which drug works” to “which drug for which patient,” with combination therapy representing the logical next Frontier given the multifactorial pathogenesis of MASH. The key pharmacologic agents discussed in the preceding sections, along with their pivotal trial data, are summarized in Table 1. This overview highlights the diverse mechanisms of action and varying stages of clinical development that define the current and emerging treatment landscape for MASLD/MASH.

**TABLE 1:** Key pharmacologic agents in MASLD/MASH (2024–2026 clinical data).

Drug	Mechanism	Phase/Status	Key trial	Key histologic outcomes (effective dose)	Target population	Approval status (2026)	Common adverse events	References
Resmetirom	THR-β agonist	Phase 3; Approved	MAESTRO-NASH	MASH resolution: 29.9% vs. 9.7%; Fibrosis ≥1-stage improvement: 25.9% vs. 14.2% (52 weeks, 100 mg)	Non-cirrhotic F2–F3 MASH	FDA 2024	Diarrhea, nausea (mild-moderate)	Harrison et al. (2024), Noureddin et al. (2025d)
Semaglutide	GLP-1 agonist	Phase 3; Approved	ESSENCE	MASH resolution: 62.9% vs. 34.3%; Fibrosis improvement: 36.8% vs. 22.4% (72 weeks, 2.4 mg)	F2–F3 MASH	FDA 2025	Nausea, diarrhea, vomiting	Sanyal et al. (2025), Bansal et al. (2026)
Tirzepatide	GLP-1/GIP agonist	Phase 3 ongoing	SYNERGY-NASH	MASH resolution: up to 62%; Fibrosis improvement: ∼51% (52 weeks, 15 mg)	F2–F3 MASH	Pending	Gastrointestinal (dose-dependent)	Caussy et al. (2025), Loomba et al. (2024a)
Efruxifermin	FGF21 analogue	Phase 3 ongoing	HARMONY; SYMMETRY	Fibrosis improvement: 49% vs. 19% (96 weeks, F2–F3, 50 mg); Trends in compensated cirrhosis	F2–F4 (compensated) MASH	Pending	Gastrointestinal (mild-moderate)	Noureddin et al. (2025a), Noureddin et al. (2025b), Harrison et al. (2025b)
Lanifibranor	Pan-PPAR agonist	Phase 3 (topline 2026)	NATiV3	IHTG reduction ∼50%; Improved insulin resistance (phase 2); Biomarker response signatures	T2DM + MASLD; Non-cirrhotic MASH	Pending (2026)	Mild GI events, modest weight gain	Barb et al. (2025), Boursier et al. (2025)
Denifanstat	FASN inhibitor	Phase 3 initiated	FASCINATE-2	NAS ≥2-point improvement: 38% vs. 16%; MASH resolution: 26% vs. 11% (52 weeks, 50 mg)	F2–F3 MASH	Pending	Dry eye (9%), alopecia (19%)—grade 1–2	Loomba et al. (2024b)
Pemvidutide	GLP-1/glucagon agonist	Phase 2b; Phase 3 planned	IMPACT	MASH resolution: 52%–58% vs. 20% (24 weeks); LFC reduction up to 68.5%	F2–F3 MASH	Pending	Gastrointestinal (mild-moderate)	Noureddin et al. (2025c), Harrison et al. (2025a)
Survodutide	GLP-1/glucagon agonist	Phase 3 ongoing	NCT04771273	MASH improvement: 43%–62% vs. 14% (48 weeks, phase 2)	F1–F3 MASH	Pending	Nausea (66%), diarrhea (49%), vomiting (41%)	Sanyal et al. (2024)
ION224	DGAT2 antisense	Phase 2	ION224-CS2	NAS ≥2-point reduction: 46%–59% vs. 19% (51 weeks, weight-independent)	F1–F3 MASH	Pending	Well-tolerated	Loomba et al. (2025)

## Clinical practice challenges and potential solutions

**FIGURE 1:**
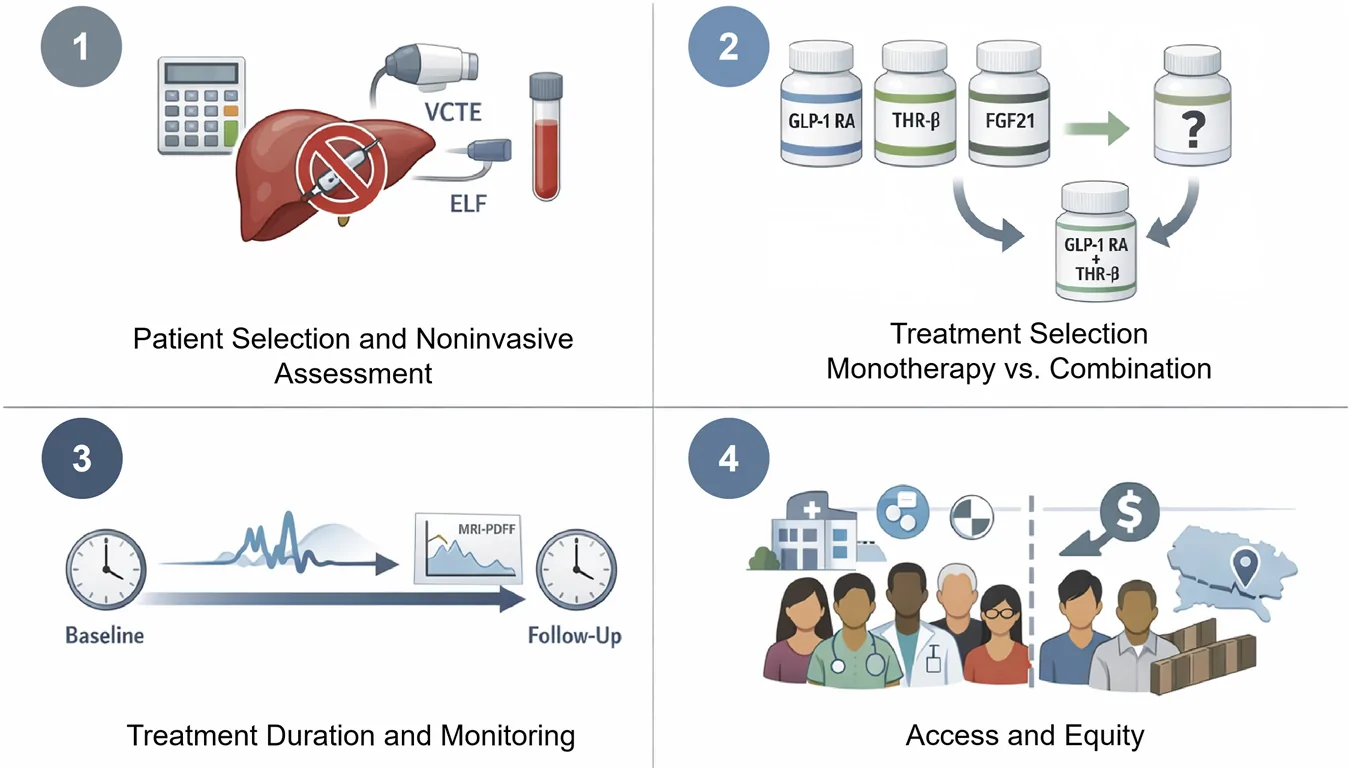
Major clinical practice challenges in lmplementing pharmacotherapy for MASLD.

The transition from clinical trials to routine practice is rarely seamless, and the integration of these new therapies presents several immediate challenges that will shape the field for the next decade. Figure 1 shows the major clinical practice challenges in implementing pharmacotherapy for metabolic dysfunction-associated steatotic liver disease (MASLD). Four interconnected hurdles—patient selection using noninvasive tests, treatment selection amid limited comparative efficacy data, determination of optimal treatment duration and monitoring strategies, and ensuring equitable access—are illustrated with corresponding pragmatic solutions. Addressing these challenges is essential for maximizing population-level benefit from emerging therapies.

Each quadrant represents one challenge, with illustrative icons and key points highlighting the primary clinical pain point and proposed pragmatic solutions. VCTE, vibration-controlled transient elastography; ELF, enhanced liver fibrosis score.

Selecting the right patient for treatment remains the most fundamental hurdle. Liver biopsy, long the imperfect reference standard, is too invasive, prone to sampling error, and impractical for the millions who need risk stratification. Validated noninvasive tests—FIB-4, vibration-controlled transient elastography (LSM by VCTE), and the ELF score—can reliably identify advanced fibrosis (F3-F4) in cross-sectional studies (Younossi et al., 2023; Riazi et al., 2022). Yet, their role in guiding treatment initiation is less settled. Regulatory acceptance of metrics like a ≥30% reduction in MRI-PDFF or a decrease in LSM as surrogate endpoints for accelerated approval has successfully de-risked drug development. However, for the practicing clinician, the thresholds that reliably predict a meaningful histologic response—and thus justify long-term pharmacotherapy—remain debated. A pragmatic, sequential screening algorithm represents a balanced approach for the clinic. All patients with MASLD and metabolic risk factors (T2DM, obesity, or multiple metabolic syndrome components) should undergo FIB-4 screening. For those with indeterminate or high risk (FIB-4 ≥1.3), evaluation proceeds to VCTE or ELF. Patients with an LSM ≥8 kPa or ELF ≥9.8—suggesting probable F2-F3 fibrosis—are appropriate candidates for a shared decision-making discussion about pharmacotherapy (Bansal et al., 2026). Biopsy, in this framework, is reserved for resolving discordant noninvasive tests, ruling out competing diagnoses, or for patients in clinical trials.

Once treatment is initiated, the clinician is immediately confronted with a dilemma of choice. With multiple agents now available and more on the horizon, head-to-head trial data to guide selection are lacking. Should therapy begin with monotherapy or a combination? If monotherapy, which agent first? If combination, which agents and when? The multifactorial pathogenesis of MASH—spanning hepatic lipogenesis, insulin resistance, inflammation, and fibrogenesis—provides a powerful mechanistic rationale for combination therapy targeting these distinct nodes simultaneously. Early-phase data are supportive: adding efruxifermin to a stable GLP-1 regimen yielded additive hepatic fat reduction without unexpected safety signals (Harrison et al., 2025b), and resmetirom’s efficacy appears independent of background GLP-1 use (Noureddin et al., 2025d). However, critical questions remain unanswered. It is not yet known whether these combinations provide additive or truly synergistic histologic improvement, whether overlapping toxicity profiles will limit tolerability, or what the optimal sequencing (initial combination vs. sequential add-on) should be. Cost-effectiveness is another unknown. In current practice, a comorbidity-guided monotherapy approach is favored for most patients. Those with overt T2DM or obesity are prioritized for GLP-1-based therapies that address both hepatic and extrahepatic manifestations, while patients with significant dyslipidemia may benefit from resmetirom’s LDL-lowering effects. For patients with high-risk features—such as F3 fibrosis, young age, or rapid progression on monitoring—or those with an inadequate response to initial monotherapy, combination therapy is a logical and necessary next step. Dedicated, well-powered combination trials are urgently needed and should be a top priority for sponsors and academic research networks.

A third major challenge is determining treatment duration and monitoring strategies. Registration trials have demonstrated histologic improvement at 52–96 weeks, but evidence-based guidance on what comes next is lacking. When is it safe to stop therapy? When should re-biopsy occur? And how should noninvasive tests be integrated into longitudinal care? Long-term extension studies like MAESTRO-NASH-OUTCOMES and the ESSENCE extension will begin reporting hard outcomes—progression to cirrhosis, decompensation, HCC, and mortality—in 2027 and 2028 (Harrison et al., 2024; Sanyal et al., 2025). Until then, histologic improvement at 52–72 weeks serves as the best surrogate predictor of long-term benefit. To navigate this uncertainty, a structured monitoring approach is recommended. All treated patients should undergo a comprehensive baseline NIT assessment (VCTE, ELF, and MRI-PDFF where available), with repeat testing every 6–12 months. A significant on-treatment improvement—for instance, an LSM reduction of ≥20% or a ≥30% decrease in MRI-PDFF—provides strong support for continuing therapy. Conversely, a lack of response or evidence of progression should prompt a re-biopsy to fully assess histologic status and guide a treatment modification, whether a dose adjustment, switching agents, or adding a second drug. The question of when to stop therapy is the most difficult. Treatment discontinuation might be considered in patients who achieve sustained normalization of NITs and substantial weight loss, but data supporting this approach are nonexistent. In this context, shared decision-making with the patient, grounded in a clear discussion of the uncertainties, is essential.

Finally, the promise of this new therapeutic era will be hollow if it is not matched by equitable access. These novel agents carry substantial costs, and reimbursement policies too often lag behind regulatory approvals. More concerning are the disparities already documented in real-world cohorts like TARGET-NASH, which show that minority populations and those with lower socioeconomic status are less likely to receive specialty care, undergo appropriate NIT assessment, or even be considered for pharmacotherapy. These disparities threaten to exacerbate existing inequities in liver-related outcomes. Addressing this requires coordinated action from multiple stakeholders. Pharmaceutical companies must implement equitable pricing and robust patient assistance programs. Payers need to develop timely, evidence-based coverage policies that do not create bureaucratic hurdles. Professional societies have a role in advocating for the inclusion of underrepresented populations in clinical trials and real-world evidence generation. And at the bedside, clinicians must be aware of and actively counter implicit biases in treatment decisions. Innovation without equitable access is, at best, an incomplete victory.

## Future directions: toward precision medicine

The therapeutic revolution in MASLD, while transformative, simultaneously illuminates critical evidence gaps and defines the roadmap toward precision medicine. Long-term data from ongoing extension studies (MAESTRO-NASH-OUTCOMES for resmetirom, ESSENCE for semaglutide, and HARMONY for efruxifermin) are eagerly awaited to confirm that histologic improvement translates into reduced incidence of cirrhosis, decompensation, hepatocellular carcinoma, and mortality, while head-to-head comparative effectiveness and rationally designed combination trials are urgently needed to guide initial agent selection and define optimal multi-target strategies in a disease of multifactorial pathogenesis (Harrison et al., 2023; Zhou et al., 2025). A parallel and equally pressing priority is the development and validation of biomarker-guided precision medicine approaches to move beyond a one-size-fits-all paradigm; emerging data, such as the lanifibranor biomarker signatures (E1 score incorporating baseline adiponectin and ferritin; changes in matrix metalloproteinase 9 and transferrin, with additional models including aminotransferases) to predict histologic response with an AUC of 0.80–0.81, offer a compelling proof-of-concept that similar proteomic and transcriptomic platforms under investigation could soon enable clinicians to match patients with the agent most likely to confer benefit (Boursier et al., 2025). Crucially, this pharmacological progress must be contextualized within the systemic nature of the disease, mandating dedicated assessment of extrahepatic outcomes given that cardiovascular disease remains the leading cause of mortality; a recent meta-analysis confirms that MASLD is associated with a significantly increased risk of cardiovascular events, with the magnitude of risk varying by fibrosis stage—individuals without advanced fibrosis exhibit a 60% increased risk of combined CVD events (HR 1.60, 95% CI 1.30–1.97), while those with advanced fibrosis face a nearly 80% elevated risk (HR 1.79, 95% CI 1.09–2.92), underscoring that cardiovascular risk intensifies with progressive liver disease (Bhatia et al., 2026). While GLP-1-based therapies have established cardioprotective effects in type 2 diabetes, analogous outcome data for THR-β agonists, FGF21 analogues, and other novel mechanisms are conspicuously lacking and must be generated through dedicated cardiovascular outcome trials or robust observational studies (Lee et al., 2025; Chui et al., 2024). Ultimately, the successful translation of these scientific advances into population-level benefit will depend on overcoming the final common pathway of implementation, requiring the development of integrated, multidisciplinary care models that co-locate or coordinate hepatology, endocrinology, cardiology, nutrition counseling, and behavioral support (El-Kassas et al., 2024), alongside dedicated implementation science research to identify and surmount barriers to equitable, evidence-based care delivery across the diverse practice settings where the growing population of MASLD patients are managed (Lionis et al., 2024).

## Conclusion

The years 2024–2026 have heralded a transformative era in MASLD management, marked by the approval of resmetirom and semaglutide—the first disease-modifying therapies for MASH—and an unprecedented pipeline of mechanistically diverse agents poised to further expand options. These advances, built on decades of recognising fibrosis as the dominant prognostic driver and the limitations of lifestyle intervention alone, decisively shift the paradigm from therapeutic nihilism to proactive, risk-stratified intervention in patients with moderate-to-advanced disease. Critically, pharmacotherapy does not supplant but amplifies lifestyle modification, with weight loss-enhancing agents overcoming physiological barriers to sustained behavioural change and yielding superior histologic responses. Comorbidity-guided initial selection, coupled with emerging evidence supporting combination approaches targeting complementary pathways, promises personalised, multi-target strategies tailored to the multifactorial pathogenesis of MASH. Nevertheless, substantial challenges remain: validating noninvasive thresholds for treatment initiation, establishing long-term clinical outcomes beyond surrogate endpoints, defining optimal sequencing and duration of therapy, and ensuring equitable global access amid high costs and systemic disparities. Addressing these through dedicated comparative and combination trials, biomarker-driven precision medicine, cardiovascular outcome studies, and integrated multidisciplinary care models will be essential to translate histologic gains into reduced morbidity and mortality.

Ultimately, by embedding new pharmacologic tools within a non-negotiable foundation of lifestyle optimisation, the field is poised to mitigate the escalating burden of MASLD, provided innovation is matched by rigorous implementation and inclusive delivery.

## Search strategy and selection criteria

This perspective article is based on a focused review of published clinical trials and translational studies that have shaped the recent paradigm shift in the pharmacological management of metabolic dysfunction-associated steatohepatitis (MASH). A literature search was performed in PubMed, clinicaltrials.gov, and the Cochrane Library from 1 January 2020, to 1 May 2026. This broad temporal frame was chosen to capture the foundational evidence leading up to and including the transformative 2024–2026 period. The search combined terms related to disease (“metabolic dysfunction-associated steatohepatitis”, “MASH”, “MASLD”, “nonalcoholic steatohepatitis”, “NASH”) with terms for pharmacologic interventions (“resmetirom”, “semaglutide”, “tirzepatide”, “efruxifermin”, “lanifibranor”, “denifanstat”, “FGF21”, “GLP-1 receptor agonist”, “PPAR agonist”, *etc.*) and trial phases (“phase 2”, “phase 3”). We prioritized pivotal, late-phase randomized controlled trials and their long-term extensions that reported histological outcomes or validated surrogate endpoints. Key guideline documents from AASLD and EASL were also included to provide clinical context. Reference lists of identified articles were manually screened for additional relevant studies. Given the nature of this article as a clinical perspective rather than a systematic review, the selection of literature is inherently selective and oriented toward trials informing imminent clinical practice and the future therapeutic landscape.

## Data Availability

The original contributions presented in the study are included in the article/Supplementary Material, further inquiries can be directed to the corresponding author.
